# Changes in the physicochemical parameters and microbial community of a new cultivar blue wheat cereal wholemeal during sourdough production

**DOI:** 10.3389/fmicb.2022.1031273

**Published:** 2022-12-08

**Authors:** Elena Bartkiene, Vytaute Starkute, Egle Zokaityte, Dovile Klupsaite, Ernestas Mockus, Modestas Ruzauskas, Vadims Bartkevics, Anastasija Borisova, João Miguel Rocha, Fatih Ozogul, Zilvinas Liatukas, Vytautas Ruzgas

**Affiliations:** ^1^Institute of Animal Rearing Technologies, Lithuanian University of Health Sciences, Kaunas, Lithuania; ^2^Department of Food Safety and Quality, Lithuanian University of Health Sciences, Kaunas, Lithuania; ^3^Faculty of Veterinary Medicine, Institute of Microbiology and Virology, Lithuanian University of Health Sciences, Kaunas, Lithuania; ^4^Institute of Food Safety, Animal Health and Environment (BIOR), Riga, Latvia; ^5^Laboratory for Process Engineering, Environment, Biotechnology and Energy, Faculty of Engineering, University of Porto, Porto, Portugal; ^6^Associate Laboratory in Chemical Engineering, Faculty of Engineering, University of Porto, Porto, Portugal; ^7^Department of Seafood Processing Technology, Faculty of Fisheries, Çukurova University, Adana, Turkey; ^8^Institute of Agriculture, Lithuanian Research Centre for Agriculture and Forestry, Akademija, Lithuania

**Keywords:** blue wheat, fermentation, sourdough, amino acids, gamma-aminobutyric acid, biogenic amines, volatile compounds

## Abstract

Changes in the characteristics of a new cultivar (DS8472-5) of blue wheat during wholemeal fermentation with *Pediococcus acidilactici* (LUHS29), *Liquorilactobacillus uvarum* (LUHS245), and *Lactiplantibacillus plantarum* (LUHS122), including acidity, microbiological and chromaticity parameters, free amino acid (FAA), gamma-aminobutyric acid (GABA), and biogenic amine (BA) contents, macro- and micro-element concentrations and fatty acid (FA) and volatile compounds (VC), were evaluated. In addition, a metagenomic analysis was performed. The lactic acid bacteria (LAB) strains used for fermentation was a significant factor in wholemeal fermentation sample pH, redness (a*) and LAB counts (*p* ≤ 0.05). In most of the samples, fermentation increased the FAA content in wheat wholemeal, and the highest concentration of GABA was found in DS8472-5 LUHS122 samples. Phenylethylamine (PHE) was found in all wheat wholemeal samples; however, spermidine was only detected in fermented samples and cadaverine only in DS8472-5 LUHS122. Fermented samples showed higher omega-3 and omega-6 contents and a higher number and variety of VC. Analysis of the microbial profile showed that LAB as part of the natural microbiota present in cereal grains also actively participates in fermentation processes induced by industrial bacterial cultures. Finally, all the tested LAB were suitable for DS8472-5 wheat wholemeal fermentation, and the DS8472-5 LUHS122 samples showed the lowest pH and the highest LAB viable counts (3.94, 5.80°N, and 8.92 log_10_ CFU/g, respectively).

## Introduction

Colored wheat cereals rich in anthocyanins have gained attention due to their health benefits (Gupta et al., 2021; Saini et al., 2021; Sharma et al., 2021, 2022). In addition, they are promising ingredients for the development of whole grain functional foods. It was reported that grasses, sprouts, whole plants and cereals of colored wheat possess anti-inflammatory and antimicrobial properties and confer other human health benefits (Mbarki et al., 2018; Paznocht et al., 2018; Sharma et al., 2018, 2020, 2021; Sytar et al., 2018; Gupta et al., 2021; Saini et al., 2021). The blue color of cereal is due to localization of anthocyanins in the aleurone layer (Garg et al., 2016). Despite the growing trend toward nutrition-rich, value-added foods, little is presently known about the nutritional background of colored wheat cereal (Tian et al., 2018) or about the changes that could occur during technological processes such as sourdough bread preparation.

Sourdough bread has been prepared in many countries for thousands of years (Wang et al., 2022), and products prepared with sourdough possess superior sensory properties to those prepared with regular yeast (baker’s yeast), because they contain a higher concentration and variety of volatile compounds (VC) (Gänzle and Zheng, 2019), among many other beneficial functional and technological traits (Rizzello et al., 2019).

The main microorganisms in sourdough are lactic acid bacteria (LAB) and yeast (Ferraz et al., 2021). It is usually difficult to predict the quality of the end product of spontaneous sourdough fermentation. For this reason, nowadays, pure LAB strains with well-known properties are used for sourdough bread preparation. The use of selected strains fulfills the need for a controlled, optimized and standardized fermentation process (Zhang et al., 2018). According to De Vuyst et al. (2014), the benefits of sourdough over yeast bread fermentations are better technological, nutritional and organoleptic properties and a longer bread shelf-life. However, the influence of sourdough on bread quality is related to several factors, such as the sourdough preparation process and the microorganisms involved (Chavan and Chavan, 2011). The LAB viable count in sourdoughs reaches, on average, 10^8^ CFU/g (Hammes et al., 2005). However, we hypothesized, that these numbers can vary with the use of colored wheat for sourdough preparation, due to the antimicrobial properties of anthocyanins. Wheat cereal grains are amber in color, however, new colored wheat grain varieties gaining interest due to desirable functional compounds—anthocyanins (Garg et al., 2016). These flavonoid structure compounds possess antioxidant, anti-inflammatory (Sharma et al., 2018), and antimicrobial properties (Enaru et al., 2021). It was reported, that small molecules, obtained after anthocyanins bio-degradation, impact the growth of colonic beneficial microbiota (Espley et al., 2014). For this reason, colored wheat grains has additional health benefits (Kapoor et al., 2022).

Baking sourdough is a complex biological system, and many endogenous (the composition of the fermentable substrate, the original microbiota of the cereal, etc.) and exogenous (temperature, duration of fermentation, etc.) factors can affect its final characteristics. The choice of a specific LAB strain is an important factor toward the control of sourdough and bread preparation. The characteristics of LAB are studied by evaluating their influence on sourdough and bread quality parameters (Suo et al., 2021). However, different LAB can vary in their influence on free amino acid (FAA) formation in sourdoughs, as well as on the occurrence of biogenic amines (BA). As the latter, when present in high concentrations, are a potential public health concern (Simon Sarkadi, 2017, 27), their control during sourdough preparation is also an important challenge. In addition to FAA, during sourdough preparation, the fatty acid (FA) profile can change, and both FAA and FA can be involved in volatile compound (VC) formation. During fermentation, valuable compounds such gamma-aminobutyric acid (GABA) can be obtained. For this reason, in this study, three different LAB strains were tested in sourdough preparation from wholemeal fermentation of new cultivars of blue wheat DS8472-5, and the most appropriate one was selected.

This study aimed to evaluate changes in the characteristics of wheat cereal wholemeal (WCW) of a new cultivar (DS8472-5) of blue wheat during fermentation with *Pediococcus acidilactici*-LUHS29, *Liquorilactobacillus uvarum*-LUHS245 and *Lactiplantibacillus plantarum*-LUHS122 strains. To this end, the following parameters were analyzed: acidity, microbiological and chromaticity parameters; FAA, GABA and BA contents; macro- and microelement concentrations; and FA and VC profiles of the non-treated and fermented WCW. In addition, a metagenomic analysis of fermented and non-fermented WCW was performed to better understand the microbiological changes obtained during sourdough preparation from the new cereal cultivar DS8472-5.

## Materials and methods

### Wheat and lactic acid bacteria strains used for fermentation

**FIGURE 1:**
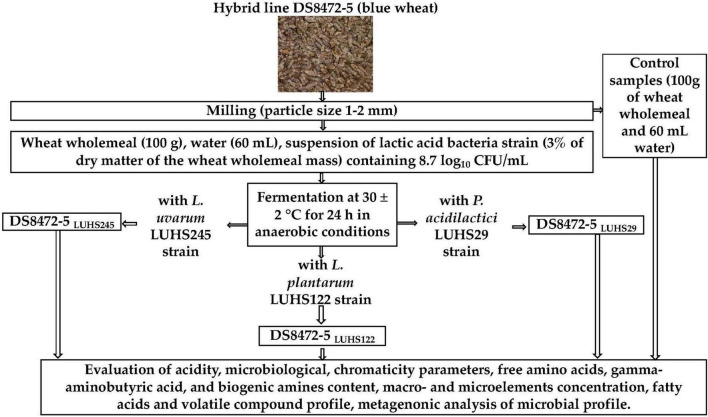
Schematic representation of the experimental design (DS847*2-5*—new cultivar of blue wheat; CFU, colony forming units; LUHS29, treated with Pediococcus acidilactici LUHS29; LUHS245, treated with Liquorilactobacillus uvarum LUHS245; LUHS122, treated with Lactiplantibacillus plantarum LUHS122).

The general scheme of the experimental procedure is shown in Figure 1. The new cultivar of blue wheat cereal DS8472-5 was provided by the LAMMS (Institute of Agriculture, Lithuanian Research Centre for Agriculture and Forestry, Akademija, Këdainiai distr., Lithuania). A description of the field trials is given in Supplementary material 1. WCW was prepared by milling wheat grain (moisture content of the wheat was 14%) with a Laboratory Mill 120 (Perten Instruments AB, Stockholm, Sweden) to a particle size of 1–2 mm.

The LAB strains *Liquorilactobacillus uvarum* (strain LUHS245), *Pediococcus acidilactici* (strain LUHS29), and *Lactiplantibacillus plantarum* (strain LUHS122) were applied for WCW fermentation. The LAB strains, which possess antibacterial and antifungal properties (Bartkiene et al., 2020), as well as LUHS245 and LUHS29 good technological characteristics for wheat bread preparation (Bartkiene et al., 2017b,2021a) and LUHS245 and LUHS122 properties to increase safety parameters of wheat bran (Bartkiene et al., 2021b), were obtained from the Department of Food Safety and Quality (Lithuanian University of Health Sciences, Kaunas, Lithuania). Before the experiments, LAB strains were cultured in de Man–Rogosa–Sharpe (MRS) broth (Oxoid Ltd., Hampshire, UK) at 30 ± 2°C for 24 h before use for the fermentation trials of WCW. The WCW, water and a suspension of the LAB strain (3% of dry matter of the WCW mass) containing 8.7 log_10_ CFU/mL were fermented in a thermostat chamber at 30 ± 2°C for 24 h. For 100 g of WCW, 60 mL of water was used (the sourdough yield 160). Non-fermented WCW samples (mixture of 100 g of WCW and 60 mL of water) were analyzed as controls.

### Chromaticity, acidity, and microbiological characteristics of wheat cereal wholemeal samples

The color coordinates (L*—lightness; a*—redness; b*—yellowness) of WCW were analyzed using ChromaMeter CR-400 (Konica Minolta, Tokyo, Japan) and expressed in National Bureau of Standards (NBS) units. The pH of WCW was measured using a pH electrode (PP-15; Sartorius, Goettingen, Germany).

The total titratable acidity (TTA) was determined for a 10 g sample homogenized with 90 mL of distilled water and expressed as the volume (mL) of 0.1M NaOH solution needed for reaching pH 8.2 (TTA assessed in the Neiman degrees,°N) (Bartkiene et al., 2017a).

For the evaluation of LAB count, 10 g of sample were homogenized with 90 mL of saline (9 g/L NaCl solution). Serial dilutions of 10^–4^–10^–8^ with saline were used for sample preparation. Sterile MRS agar (CM0361, Oxoid) of 5 mm thickness was used for bacterial growth (Man, Rogosa, Sharpe) on Petri plates. The plates were separately seeded with the sample suspension using sowing in surface and were incubated under anaerobic conditions at 30°C for 72 h. The number of bacterial colonies was calculated and expressed as a decimal log of colony forming units per gram of sample (CFU/g).

### Analysis of free amino acids in wheat cereal wholemeal samples

Sample preparation and dansylation were performed according to the method of Cai et al. (2010), with some modifications, which are given in Supplementary material 2.1.

### Determination of biogenic amine content in wheat cereal wholemeal samples

The extraction and determination of BA in non-fermented and fermented WCW followed the procedures developed by Ben-Gigirey et al. (1999). Derivatization of samples was performed with dansyl chloride. The standards of BA (cadaverine, histamine, PHE, putrescine, spermidine, spermine, tryptamine, and tyramine) were prepared by dissolving known amount of each BA in 20 mL of deionized water. An Agilent 1200 HPLC (Carlsbad, CA, USA) equipped with a diode-array detector (DAD) and Chemstation LC software was employed. A Chromolith C18 HPLC column (100 mm × 4.6 mm × 4 mm, Merck KGaA/EMD Chemicals, Darmstadt, Germany) was used. The detection limit for BA was 0.1 mg/kg.

### Analysis of micro- and macro-elements

For micro- and macro-element evaluation, an Agilent 7700x inductively coupled plasma mass spectrometry (ICP-MS) (Agilent Technologies, Tokyo, Japan) and *MassHunter WorkStation* software version B.01.01 (Agilent Technologies, Tokyo, Japan) were used. Sample preparation for ICP-MS analysis was carried out as described by Bartkiene et al. (2016).

### Analysis of wheat cereal wholemeal fatty acid profiles

The FA profile of WCW samples was analyzed using a QP2010 gas chromatograph with a mass spectrometer (GC-MS) (Shimadzu, Japan). The fatty acid methyl ester (FAME) concentration was determined using a calibration curve, and the results were expressed as a percentage of the total FA in the sample. The method description is given in Supplementary material 2.2.

### Evaluation of the volatile compound profiles of non-treated and fermented wheat cereal wholemeal samples

The VCs of the WCW samples were analyzed by GC-MS. For this purpose, a solid phase microextraction (SPME) device with Stableflex™ fiber coated with a 50 μm PDMS-DVB-Carboxen™ layer (Supelco, USA) was used. The method description is given in Supplementary material 2.3.

### Metagenomic analysis of fermented and non-fermented wheat cereal wholemeal samples

Non-fermented and fermented WCW samples (5 g of each sample) were collected for bacterial profiling analysis. A Quick-DNA Fecal/Soil Microbe Kit (Zymo Research, Irvine, CA, USA) was used for total DNA extraction according to the manufacturer’s instructions. The initial quantity and quality of the DNA were controlled using a Nano Drop 2000 spectrophotometer (Thermo Fisher, Waltham, MA, USA). Metagenomic libraries, sequencing of the 16S rRNA gene and sequence analysis were performed in an independent service laboratory (Novogene, Cambridge, UK). The target region was V3-V4 and the primers used were 341F (CCTAYGGGRBGCASCAG) and 806R (GGACTACNNGGGTATCTAAT) with barcodes. The PCR products with proper size were selected by 2% agarose gel electrophoresis. Same amount of PCR products from each sample was pooled, end-repaired, A-tailed and further ligated with Illumina adapters. Libraries were sequenced on a paired-end Illumina platform to generate 250 bp paired-end raw reads. The library was checked with Qubit and real-time PCR for quantification and bioanalyzer for size distribution detection. Paired-end reads were assigned to samples based on their unique barcodes and truncated by cutting off the barcode and primer sequences. Paired-end reads were merged using FLASH (V1.2.7) tool, which was designed to merge paired-end reads when at least some of the reads overlap the read generated from the opposite end of the same DNA fragment, and the splicing sequences were called raw tags. Quality filtering on the raw tags were performed under specific filtering conditions to obtain the high-quality clean tags according to the Qiime (V1.7.0) quality controlled process. The tags were compared with the reference database (SILVA138) using UCHIME algorithm to detect chimera sequences. Then the chimera sequences were removed and the Effective Tags obtained. Sequences analysis were performed by Uparse software (Uparse v7.0.1090) using all the effective tags. Sequences with ≥ 97% similarity were assigned to the same OTUs. When analyzing bacterial diversity in the samples, OTUs of algae and plants (DNA of chloroplasts) were omitted.

### Statistical analysis

Three parallel samples for fermentations were performed. Microbiological data were expressed as average (*n* = 5) ± standard error (SE), whereas physicochemical data were expressed as average (*n* = 3) ± SE. One-way analysis of variance (ANOVA) was used to evaluate an influence of the used LAB on WCW parameters. The linear Pearson’s correlation coefficients and their significance were calculated using the statistical package SPSS for Windows [v28.0.1.0 (142), SPSS, Chicago, Illinois, USA], correlation strength was interpreted according to Evans (1996). The results were recognized as statistically significant at *p* ≤ 0.05.

## Results and discussion

### Chromaticity, acidity, and microbiological characteristics of wheat cereal wholemeal samples

**FIGURE 2:**
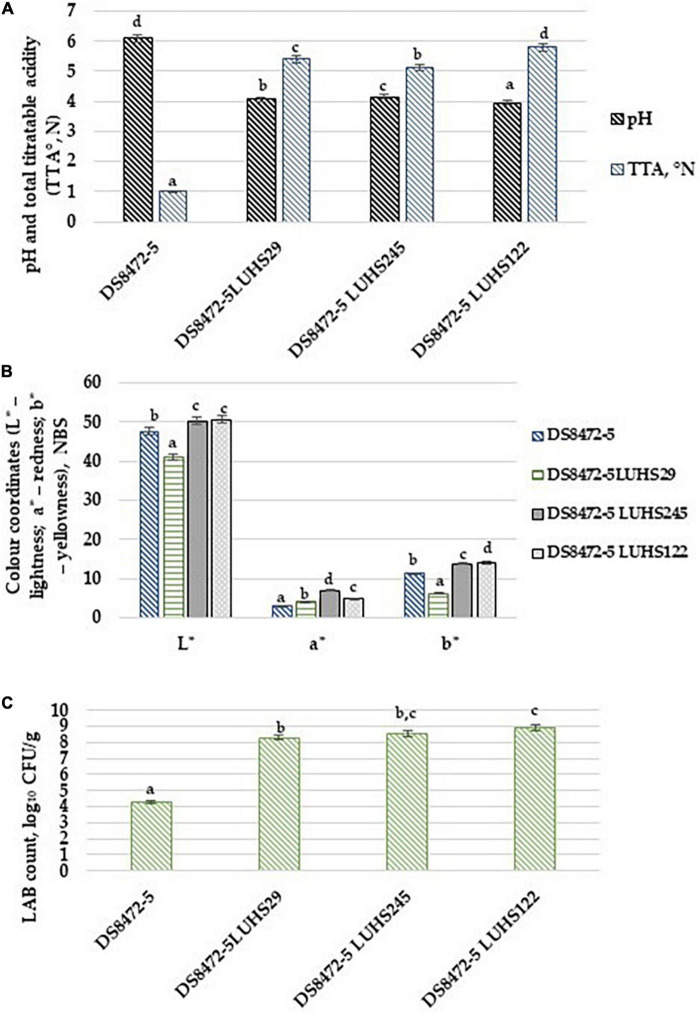
**(A)** Acidity, **(B)** chromaticity and **(C)** microbiological characteristics of wheat cereal wholemeal (WCW) (DS8472-5) samples. LAB, lactic acid bacteria; CFU, colony forming units; L*, lightness; a*, redness; b*, yellowness. LUHS29, treated with *Pediococcus acidilactici* LUHS29*;* LUHS245, treated with *Liquorilactobacillus uvarum* LUHS245*;* LUHS122, treated with *Lactiplantibacillus plantarum* LUHS122. Physicochemical parameters presented as the mean (*n* = 3) ± standard error (SE). The data on lactic acid bacterial viable counts presented as the mean of (*n* = 5) ± SE. ^a–d^Means with different letters in column are significantly different (*p* ≤ 0.05).

The chromaticity, acidity and microbiological characteristics of the WCW samples are shown in Figures 2A–C, respectively). Regarding the comparison of acidity parameters, fermentation with selected LAB strains decreased the pH of WCW samples and increased TTA. Moreover, the lowest pH as well as the highest TTA were obtained in WCW fermented with strain *Lp. plantarum* LUHS122 (3.94 and 5.80°N, respectively). However, a significant correlation between the sample pH and TTA was not found. The LAB count in fermented WCW samples was, on average, 8.59 log_10_ CFU/g, and the LAB count in samples showed, as expected, a very strong negative correlation with the sample pH (*r* = -0.932, *p* = 0.0001).

The decrease in pH and increase in TTA are the most important changes that occurs during sourdough preparation (Wang et al., 2022), and these changes depend on the parameters of the fermentable substrate, the process conditions and the types of microorganisms involved in fermentation (Clarke et al., 2003). According to Sadeghi et al. (2019), the dietary fiber in cereal wholemeal affects microbial metabolism by influencing TTA and could be attributed to the buffering capacity.

LAB have the ability to metabolize a large variety of carbohydrates, including complex indigestible polymers (Makarova et al., 2006). It was reported, that the overall efficacy of LAB carbohydrate metabolism is related with their capacity to excrete enzymes (Filannino et al., 2018). The lowest pH of the samples fermented with *Lp. plantarum* LUHS122 strain, can be explained by previous findings, that, although LAB have a limited cellulosic machinery, the *Lp. plantarum* WCFS1 genome harbors a putative endoglucanase and eleven genes encoding for phospho-β-glycosidases activities (Siezen et al., 2012; Zhao et al., 2020). However, further studies are needed to indicate enzymes excretion efficiency of the tested LAB strains.

When comparing sample chromaticity parameters, the lowest lightness (L*) and yellowness (b*) were found in the WCW samples fermented with strain *P. acidilactici* LUHS29 (40.9 and 6.12 NBS, respectively). However, non-fermented WCW samples showed the lowest redness (a*) (3.03 NBS). The WCW samples L* and b* parameters showed moderate positive correlations with TTA of samples (*r* = 0.747, *p* = 0.005 and *r* = 0.635, *p* = 0.026, respectively). A moderate negative correlation between the a* coordinates and pH of the samples was found (*r* = −0.717, *p* = 0.009), as well as a moderate positive correlation between the a* coordinates and LAB counts of the samples (*r* = 0.635, *p* = 0.026).

The stability of anthocyanins is related to their chemical structure, along with the processing temperature, pH and exposure to light, oxygen, enzymes, etc. (Lim et al., 2011; Riaz et al., 2016). They can be partially degraded by the combined action of cellular and environmental factors (Lim et al., 2011; Riaz et al., 2016). Anthocyanidin is highly reactive due to the electron-deficient flavylium cation structure (Castañeda-Ovando et al., 2009; Lim et al., 2011; Riaz et al., 2016). The deglycosylated form is particularly vulnerable to water; however, glycosylated and acylated anthocyanins are more stable (Castañeda-Ovando et al., 2009; Riaz et al., 2016). The test of between-subject effects showed that the LAB strain used for fermentation was a significant factor in WCW sample pH (*p* = 0.0001), a* coordinates (*p* = 0.001) and LAB counts (*p* = 0.001).

### Free amino acid, γ-aminobutyric acid, and biogenic amine contents in wheat cereal wholemeal samples

**FIGURE 3:**
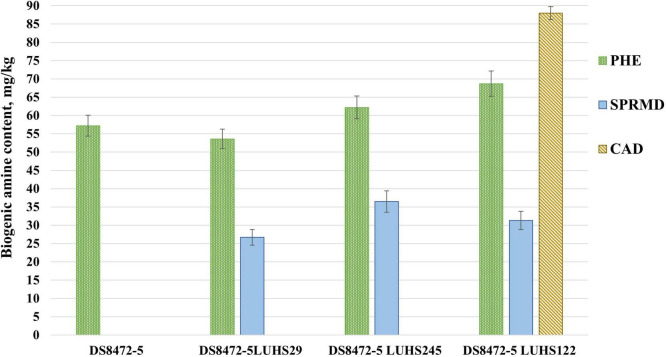
Biogenic amine (BA) concentration (mg/kg) in wheat cereal wholemeal (WCW) (DS8472-5) samples. LUHS29, treated with *Pediococcus acidilactici* LUHS29; LUHS245, treated with *Liquorilactobacillus uvarum* LUHS245; LUHS122, treated with *Lactiplantibacillus plantarum* LUHS122. PHE, phenylethylamine; CAD, cadaverine; SPRMD, spermidine. Results presented as the mean (*n* = 3) ± standard error (SE). ^a,b^Means with different letters in column are significantly different (*p* ≤ 0.05).

The FAA and γ-aminobutyric acid (GABA) contents are shown in Table 1, and biogenic amine (BA) contents in WCW samples are given in Figure 3. When comparing the arginine concentration in WCW samples, the highest content was established in WCW treated with *Liq. uvarum* LUHS245 and *Lp. plantarum* LUHS122 (on average, 1.21 μmol/g). The LAB strain used for fermentation was a significant factor on the arginine content in WCW samples (*p* = 0.0001). In addition to other physiological benefits, arginine enhances immunity and shows anti-infective and antioxidative characteristics (Wu et al., 2021).

**TABLE 1:** Free amino acids (FAA) and gamma-aminobutyric acid (GABA) content in wheat cereal wholemeal (WCW) (DS8472-5) samples.

Compound name	DS8472-5	DS8472-5_LUHS29_	DS8472-5_LUHS245_	DS8472-5_LUHS122_
	
	Free amino acids and GABA concentration, μmol/g			
Arginine	0.471 ± 0.038^b^	0.141 ± 0.011^a^	1.28 ± 0.12^c^	1.14 ± 0.11^c^
Glutamine	0.772 ± 0.041^d^	0.688 ± 0.037^c^	0.530 ± 0.042^b^	0.112 ± 0.010^a^
Serine	0.519 ± 0.026^b^	0.514 ± 0.033^b^	0.282 ± 0.017^a^	0.663 ± 0.029^c^
Aspartic acid	1.50 ± 0.12^c^	2.58 ± 0.19^d^	0.993 ± 0.053^a^	1.08 ± 0.09^a^
Glutamic acid	0.873 ± 0.032^c^	1.28 ± 0.11^d^	0.555 ± 0.043^b^	0.303 ± 0.021^a^
Threonine	0.416 ± 0.030^a^	0.938 ± 0.045^c^	0.543 ± 0.036^b^	0.522 ± 0.028^b^
Glycine	0.650 ± 0.021^a^	1.77 ± 0.15^c^	1.17 ± 0.14^b^	2.04 ± 0.18^d^
Alanine	1.65 ± 0.14^b^	3.32 ± 0.24^c^	1.34 ± 0.11^a^	3.92 ± 0.29^d^
Proline	0.403 ± 0.035^a^	1.20 ± 0.11^c^	0.864 ± 0.052^b^	1.53 ± 0.12^d^
Methionine	<0.020	0.393 ± 0.028^b^	0.310 ± 0.025^a^	0.455 ± 0.036^c^
Valine	0.500 ± 0.042^a^	1.52 ± 0.12^c^	0.939 ± 0.063^b^	2.38 ± 0.17^d^
Phenylalanine	0.177 ± 0.12^a^	0.863 ± 0.052^b^	0.795 ± 0.031^b^	1.57 ± 0.09^c^
Leucine/Isoleucine	0.406 ± 0.020^a^	3.55 ± 0.29^c^	2.47 ± 0.22^b^	4.55 ± 0.32^d^
Lysine	0.322 ± 0.024^a^	2.07 ± 0.14^c^	1.51 ± 0.10^b^	1.73 ± 0.15^b^
Histidine	0.121 ± 0.018^b^	0.227 ± 0.020^d^	0.162 ± 0.011^c^	0.019 ± 0.002^a^
Tyrosine	0.110 ± 0.008^a^	0.588 ± 0.029^b^	0.581 ± 0.035^b^	0.999 ± 0.042^c^
GABA	3.72 ± 0.26^b^	4.13 ± 0.31^c^	2.29 ± 0.17^a^	12.3 ± 0.11^d^

LUHS29—treated with *Pediococcus acidilactici* LUHS29; LUHS245—treated with *Liquorilactobacillus uvarum* LUHS245; LUHS122—treated with *Lactiplantibacillus plantarum* LUHS122; GABA—gamma-aminobutyric acid. Mean values (*n* = 3) ± standard error (SE).

^a–d^Means with different letters in column are significantly different (*p* ≤ 0.05).

The highest content of glutamine was found in control samples (10.9, 31.3, and 85.5% higher, in comparison with DS8472-5 LUHS29, DS8472-5 LUHS245, and DS8472-5 LUHS122 samples, respectively). The LAB strain used for fermentation was a significant factor on the glutamine content in WCW (*p* = 0.002). Glutamine supports the gut microbiome and gut mucosal wall integrity and modulates inflammatory responses (Deters and Saleem, 2021). The lowest serine concentration was shown in DS8472-5 LUHS245 samples (0.282 μmol/g) and the highest in DS8472-5 LUHS122 samples (0.663 μmol/g). In non-fermented and DS8472-5 LUHS29 samples, the serine content was, on average, 0.517 μmol/g. The LAB strain used for fermentation was a significant factor on the serine content in WCW (*p* = 0.0001). L-serine has an essential role in a broad range of cellular functions, including protein synthesis, neurotransmission, etc. (Holeček, 2022). The highest concentration of aspartic acid was found in DS8472-5 LUHS29 samples; other fermented samples showed, on average, a 2.48-fold lower aspartic acid content. The LAB strain used for fermentation was not a significant factor with regards to the aspartic acid concentration in WCW. A deficit of glutamine and glycine and upregulation of aspartic acid are correlated with depression-like symptoms (Yang et al., 2019).

Fermentation with strain *P. acidilactici* LUHS29 increased the glutamic acid content in WCW, on average, by 31.8%; however, in WCW fermented with strains *Liq. uvarum* LUHS245 and *Lp. plantarum* LUHS122, the glutamic acid content was reduced, on average, by 36.4 and 65.3%, in comparison with non-fermented WCW. The LAB strain used for fermentation proved to be a significant factor in the glutamic acid content in WCW (*p* = 0.0001). Glutamic acid performs an important function in the prevention of allergies and infections (Nakai et al., 2022).

In all cases, higher concentrations of threonine, glycine, proline, methionine, valine, phenylalanine, leucine/isoleucine, lysine, and tyrosine were found in fermented WCW, in comparison with non-fermented ones, and the LAB strain used for fermentation was a significant factor in most of the abovementioned FAA formation (*p* ≤ 0.05). Threonine improves immune function (Gao et al., 2014; Sharf and Khan, 2022), protects the gastrointestinal mucosa, skin and gills and is involved in the production of elastin and collagen (Habte-Tsion et al., 2015). Wheat flour is deficient in the amino acids lysine and methionine (Anjum et al., 2005), and lysine is the first limiting amino acid in wheat (Pogna et al., 1994; Jood et al., 1995).

The highest content of alanine was found in DS8472-5 LUHS122 samples, and the highest content of histidine was shown in DS8472-5 LUHS29. Histidine may significantly enhance anaerobic athletic performance in humans by delaying skeletal muscle fatigue (Hoffman et al., 2006, 2008a,2008b; Stout et al., 2006; Derave et al., 2007; Hill et al., 2007; Kendrick et al., 2008). Finally, the differences in cereal AA profiles may be due to differences in the genetic makeup and environment of wheat varieties tested by different workers (Hoffman et al., 2012).

However, it should be pointed out that the protein content of wheat ranges from 7 to 15% and plays a vital role in the human diet (Ciclitira and Ellis, 1987). The protein content, along with the amino acid profile, determines the nutritional quality of wheat grains (Joye, 2019). Although the amino acid profile of wheat cereal is not balanced (Jiang et al., 2008), the processing of wheat cereal by using fermentation with selected LAB strains can result in higher-value products.

The efforts in the production of amino acids through microbial fermentation have been conducted in several studies (Sgobba et al., 2018; Chu et al., 2021). It was reported, that *Pediococcus acidilactici* Kp10 strain is suitable starter for amino acids from pineapple plant stem hydrolysates production (Chu et al., 2021). Microorganisms generally produce 20 amino acids only in the amounts needed by the cells (Ivanov et al., 2013; Genchi, 2017). In comparison *Lactobacillus* sp. and *Pediococcus* sp., the latters showed higher production of amino acids, especially lysine and methionine (Lim et al., 2019). The ability of *P. acidilactici* to produce lysine and methionine was also reported by Lee et al. (2014) and Toe et al. (2019). However, carbon source plays an important role in the biosynthesis of the amino acids in fermentable substrate (Chu et al., 2021). This study showed that the processing of wheat cereal by using fermentation with selected LAB strains can result in higher-value products, because essential amino acids concentration in fermented samples was significantly increased.

Regarding the comparison of GABA concentrations in all tested WCW samples, the highest concentration of GABA was found in DS8472-5 LUHS122 samples (3.31, 2.98, and 5.37 times higher, in comparison with non-fermented samples and those fermented with *P. acidilactici* LUHS29 and *Liq. uvarum* LUHS245 strains, respectively). GABA is a conversion product of glutamate (Le et al., 2020). If free glutamate is present as a precursor, fermented foods are good sources of GABA. The function of GABA in the treatment of neurological disorders is well known (Ting Wong et al., 2003). GABA has been reported to have diuretic and antidiabetic effects, the ability to reduce pain and anxiety (Dhakal et al., 2012) and anti-carcinogenic activity (Oh and Oh, 2004; Al-Wadei et al., 2011). For this reason, functional foods enriched with GABA have become very popular (Aoki et al., 2003; Kim et al., 2009, 100; Li et al., 2010). It was reported, that *Pediococcus pentosaceus* F01, *Levilactobacillus brevis* MRS4, *Lactiplantibacillus plantarum* H64 and C48 strains shows the ability to synthesize GABA (Verni et al., 2019). GABA enrichment of cereal-based foods through LAB fermentation was proposed by Coda et al. (2010), Dhakal et al. (2012), Diana et al. (2014) and Venturi et al. (2019). An important step toward the achievement of high GABA content is the LAB starter selection, also, wheat bran addition increases GABA production by *L. plantarum* H64 and C48 strains (Verni et al., 2019).

Proteolysis is one of the physiological properties of LAB strains, comprises proteinases, peptidases, and specific transport proteins (Kieliszek et al., 2021).

It was reported about biochemical characterization of the LAB proteolytic enzymes, but till now, information about the inhibition, and activation of proteolytic enzymes is scarce (Savijoki et al., 2006; Jensen and Ardö, 2010; Griffiths and Tellez, 2013). It was established, that the LAB can regulate the expression of their genes in response to environmental conditions (Guan et al., 2017), as well as to respond to changes in the availability of nitrogen by regulating the activity of the proteolytic system (Brown et al., 2017; Liu et al., 2017). Finally, our study showed, that the LAB strain, used for wheat wholemeal fermentation, was significant factor on most of the amino acid content in wholemeal samples, however, further studies are needed to indicate specific proteolytic activity of the tested LAB, which is responsible for separate amino acids formation.

The main BA in non-fermented and fermented WCW was PHE, and fermentation with strains *Liq. uvarum* LUHS245 and *Lp. plantarum* LUHS122 increased the PHE content in samples, on average, by 18.1%, in comparison with samples fermented with strain LUHS29 (Figure 3). Also, cadaverine was found (88.0 mg/kg) in WCW fermented with *P. acidilactici* LUHS122, and spermidine (SPRMD) was formed in all the fermented samples. Histamine, putrescine, spermine, tryptamine, and tyramine were not found in all tested samples. A moderate negative correlation was found between the spermidine content in WCW and the sample pH (*r* = -0.781, *p* = 0.003), and a moderate positive correlation was established between the spermidine content and LAB viable counts (*r* = 0.751, *p* = 0.005).

Through the activity of fermentative and contaminating microorganisms, the amino acids decarboxylation leads to the formation of BA. The content of FAA s, environmental conditions (pH and temperature), and the presence of proteases and decarboxylase-positive bacteria influence the BA occurrence in food (Gardini et al., 2016). BA can cause headache, vomiting, diarrhea, reduced blood pressure, breathing difficulties and etc. (Barbieri et al., 2019). The toxicity of BA is related with high levels but no specific legislation regulates BA levels in food, except histamine in fish, as histamine and tyramine are the most toxic ones (Ruiz-Capillas and Herrero, 2019). In our study, only PHE, CAD, and SPRMD were found in non-fermented and fermented WCW. Polyamines such as SPRMD may have positive effect on regulation of normal growth and maturation of the intestinal tract, however, higher levels have been associated with food allergies (Karayigit et al., 2020). The amino acid lysine can be converted into CAD, which is accumulated by LAB and spoilage microorganisms, especially enterobacteria and *Pseudomonas* (Barbieri et al., 2019). PHE can be formed in the same manner from phenylalanine (Montet and Ray, 2020). However, glutamine showed a moderate negative correlation with PHE (*r* = -0.779, *p* = 0.003) in this study. It was reported that arginine or ornithine, as well as putrescine, are precursors of SPRMD (Muñoz-Esparza et al., 2019). That explains the observed strong negative correlation between the arginine and SPRMD contents in WCW (*r* = -0.802, *p* = 0.002). However, CAD and SPRMD can also occur naturally in raw plant foods (Muñoz-Esparza et al., 2019).

The total BA content in rye bread was reported 168.6 nmol/g (Cipolla et al., 2007), in Japanese food products the concentration of putrescine in bread was reported, on average, 44 nmol/g (Nishibori et al., 2007).

Hernández-Macias et al. (2022) reported, that only very low putrescine, cadaverine, and spermidine contents in bread were found.

Also, BA concentration could varied within and among the cultivars or milling fractions in the selected wheats (Karayigit et al., 2020).

### Macro- and micro-element contents in wheat cereal wholemeal samples

**FIGURE 4:**
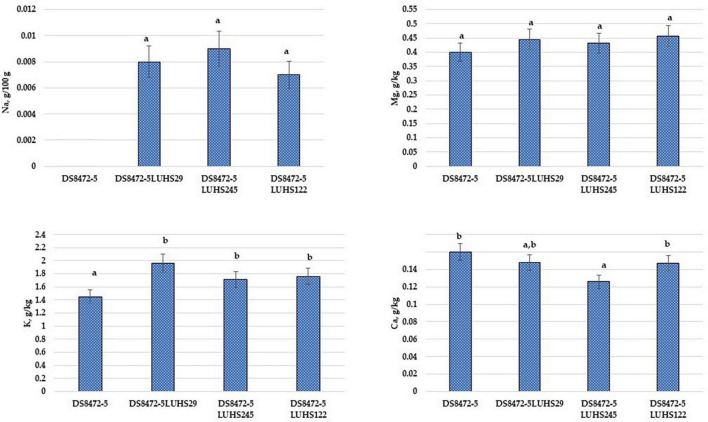
Macro-element concentrations in wheat cereal wholemeal (WCW) (DS8472-5) samples. LUHS29, treated with *Pediococcus acidilactici*; LUHS245, treated with *Liquorilactobacillus uvarum*; LUHS122, treated with *Lactiplantibacillus plantarum* LUHS122. Results presented as the mean (*n* = 3) ± SE. ^a,b^Means with different letters in column are significantly different (*p* ≤ 0.05).

The macro-element concentrations in WCW samples are shown in Figure 4. Considering that the non-fermented WCW samples were prepared by adding the same quantity of water, the difference in macro-elements between samples could be obtained by the addition and multiplication of LAB. In a comparison of Na and K concentrations, in fermented samples, these macro-elements showed a higher content (on average, by 4.0 and 1.3 times, respectively). The Mg concentration in all the tested samples was, on average, 0.433 g/kg. The lowest Ca concentration was found in with *Liq. uvarum* LUHS245 fermented samples. Despite, that significant differences in Ca content between the samples fermented with *Liq. uvarum* LUHS245 and with *P. acidilactici* LUHS29 were not found, the latter samples showed slightly higher Ca content, similar with Ca content in non-fermented and with *Lp. plantarum* LUHS122 fermented samples.

**FIGURE 5:**
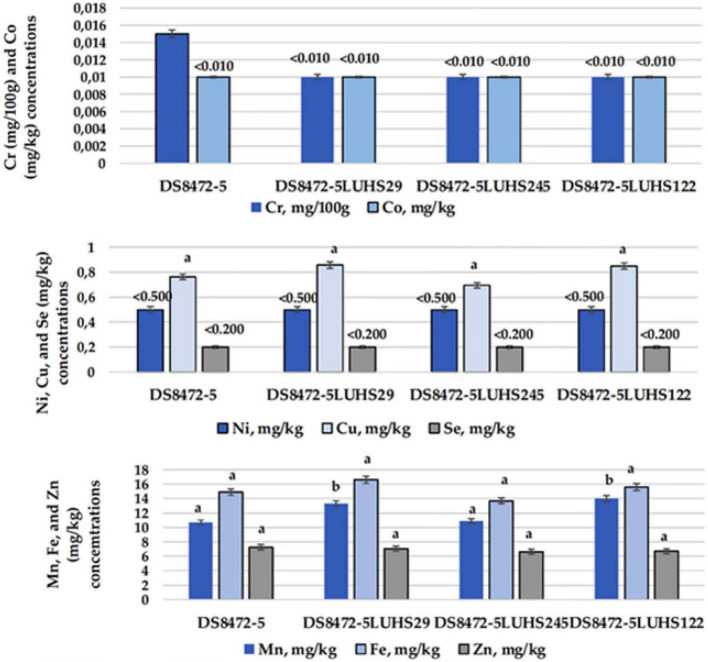
Essential micro-element concentrations in wheat wholemeal (WCW) (DS8472-5) samples. LUHS29, treated with *Pediococcus acidilactici*; LUHS245, treated with *Liquorilactobacillus uvarum*; LUHS122, treated with *Lactiplantibacillus plantarum* LUHS122. Results presented as the mean (*n* = 3) ± standard error (SE). ^a,b^Means with different letters in column are significantly different (*p* ≤ 0.05).

The essential micro-element concentrations in WCW samples are shown in Figure 5. Significant differences between Fe, Co, Ni, Cu, Zn, and Se concentrations in WCW were not established, and the concentrations of these essential micro-elements in samples were, on average, 15.2, < 0.010, < 0.500, 0.791, 6.91, and < 0.200 mg/kg, respectively. However, when comparing Cr concentrations, non-fermented WCW showed the highest Cr content (0.015 mg/100 g). A Mn content, on average, 20.9% lower, was found in non-fermented samples, as well as in WCW fermented with strain *Liq. uvarum* LUHS245, in comparison with WCW fermented with *P. acidilactici* LUHS29 and *Lp. plantarum* LUHS122.

The comparison of non-essential micro-element concentrations (As, V, Rb, Sr, Mo, Ag, Sb, Cs, TI, Cd, Ba, Pb, Al, and Li) in WCW samples unfold no significant differences, and most of these micro-element concentrations were below the detection limits, except Sr and Cd—which concentrations were, on average, 1.55 and 0.01 mg/kg, respectively.

The grain of modern high-yielding common wheat varieties is generally characterized by a low content of micro-elements (Fan et al., 2008; Cakmak et al., 2010). The scientific literature provides scant information on the differences in micro-element concentrations in various wheat cereal species; for this reason, it is very important to have a database of wheat grain composition.

Taking into consideration the popularity of wheat and wheat products, WCW products could be a very valuable source of micro- and macro-elements (Shi et al., 2008). It should be mentioned that the micro- and macro-element contents and their bioavailability can change during cereal processing (including fermentation) (Verni et al., 2019). For example, during the fermentation process, Mn^2+^, Mg^2+^, Ca^2+^, Fe^2+^, K^+^, and Na^+^ are involved in the metabolic activity of LABs (Von Wright and Axelsson, 2011) and can have a substantial influence during sourdough production. It was reported that colored wheat could have a fourfold higher organic Cr concentration, in comparison with common wheat (Guo et al., 2012). Black wheat showed significantly higher Fe, Zn, Se, Mg, Mn, Cu, K, and P concentrations, in comparison with common wheat (He and Ning, 2003), and blue wheat showed the highest Ca concentration (Tian et al., 2018). Nevertheless, it must be pointed out that fertilizers could be a significant factor in the micro- and macro-element contents in grains (Ma et al., 2018). Finally, the development of a database of new wheat cereal breed lines, as well as the evaluation of micro- and macro-element contents during technological processes, is a key issue to the efficient use of cereal in the food and/or feed industries.

Fermentation of plant-based substrates can increase minerals bioavailability (Pranoto et al., 2013; Nkhata et al., 2018). Increasing of zinc, calcium, iron and magnesium bioavailability in cereal is explained by reduced concentration of phytates (Pranoto et al., 2013; Ahmed et al., 2020). However, in overall, mineral content increases are explained by the loss of the substrate dry matter, because LAB degrade proteins and carbohydrates (Day and Morawicki, 2016). Finally, fermentation effects substrate composition, because of one compound concentration increasing, other ones reducing.

### Fatty acid profiles of wheat cereal wholemeal samples

The FA profiles of WCW samples are shown in Tables 2, 3. The dominant FA in WCW were methyl linoleate (C18:2), cis-trans-9-oleic acid methyl ester (C18:1 cis-trans), methyl palmitate (C16:0), methyl stearate (C18:0) and α-linolenic acid methyl ester (C18:3 α). Methyl tetradecanoate (C14:0) and methyl palmitoleate (C16:1) were found only in WCW samples fermented with *Liq. uvarum* LUHS245 (2.42% and 0.311% of total fat content, respectively). Also, cis-11-eicosenoic acid methyl ester (C20:1) was observed only in WCW fermented with *P. acidilactici* LUHS29 and *Liq. uvarum* LUHS245 (0.634 and 0.533% of total fat content, respectively). WCW samples fermented with *Lp. plantarum* LUHS122 showed the lowest saturated fatty acid (SFA) content (25.01% of total fat content) and, in a comparison fermented and non-fermented samples, non-fermented samples showed higher SFA concentration (by 21.7% higher in comparison with DS8472-5 LUHS29, by 1.81% higher in comparison with DS8472-5 LUHS245, and by 23.2% higher in comparison with DS8472-5 LUHS122). In all cases, fermentation reduced the monounsaturated fatty acid (MUFA) content, and the lowest MUFA content was found in WCW samples fermented with strain *Lp. plantarum* LUHS122. In contrast to MUFA, the desirable polyunsaturated fatty acid (PUFA) content in fermented samples was higher than that in non-fermented ones. Also, fermented samples showed higher ω-3 and ω-6 contents, in comparison with non-fermented ones; still, sourdough fermentation reduced the ω-9 content in WCW, and the lowest ω-9 concentration was found in samples fermented with *Lp. plantarum* LUHS122.

**TABLE 2:** Fatty acid (FA) profiles of wheat cereal wholemeal (WCW) (DS8472-5) samples.

Fatty acid concentration, % of total fat content	Wheat wholemeal samples			
	
	DS8472-5	DS8472-5LUHS29	DS8472-5 LUHS245	DS8472-5 LUHS122
C14:0	–	–	2.42 ± 0.08	–
C16:0	25.3 ± 0.2^c^	20.8 ± 0.2^a^	24.0 ± 0.1^b^	21.0 ± 0.2^a^
C16:1	–	–	0.311 ± 0.002	–
C17:0	–	–	–	–
C18:0	7.23 ± 0.12^d^	4.71 ± 0.03^b^	5.52 ± 0.16^c^	3.96 ± 0.11^a^
C18:1	19.9 ± 0.1^c^	18.4 ± 0.1^b^	18.3 ± 0.2^b^	17.4 ± 0.1^a^
C18:2	44.7 ± 0.3^a^	52.5 ± 0.2^b^	45.9 ± 0.3^a^	54.1 ± 0.2^c^
C18:3 γ	–	–	–	–
C18:3 α	2.73 ± 0.01^a^	2.93 ± 0.04^b^	2.89 ± 0.02^b^	3.38 ± 0.02^c^
C20:0	–	–	–	–
C20:1	–	0.634 ± 0.002^b^	0.533 ± 0.003^a^	–
C20:4	–	–	–	–
C22:0	–	–	–	–
C22:1	–	–	–	–

C14:0—methyl tetradecanoate; C16:0—methyl palmitate; C16:1—methyl palmitoleate; C17:0—methyl heptadecanoate; C18:0—methyl stearate; C18:1—cis-trans-9-oleic acid; C18:2—methyl linoleate; C18:3 γ—gamma-linolenic acid; C18:3 α—alfa linolenic acid; C20:0—eicosanoic acid; C20:1—cis-11-eicosenoic acid; C20:4—cis-5.8.11.14-eicosatetraenoic acid; C22:0—methyl docosanoate; C22:1—cis-13-docosenoic acid; LUHS29—treated with *Pediococcus acidilactici* LUHS29; LUHS245—treated with *Liquorilactobacillus uvarum* LUHS245; LUHS122—treated with *Lactiplantibacillus plantarum* LUHS122. Mean values (*n* = 3) ± standard error (SE). ^a–c^Means with different letters in column are significantly different (*p* ≤ 0.05).

**TABLE 3:** Fatty acid (FA) profiles of wheat cereal wholemeal (WCW) (DS8472-5) samples.

Wheat wholemeal samples	Fatty acid concentration, % from total fat content					
	
	Saturated fatty acids	Monounsaturated fatty acids	Polyunsaturated fatty acids	Omega-3	Omega-6	Omega-9
DS8472-5	32.57	19.99	47.44	2.73	44.71	19.99
DS8472-5_LUHS29_	25.49	19.08	55.43	2.93	52.50	19.08
DS8472-5_LUHS245_	31.98	19.14	48.87	2.89	45.99	19.14
DS8472-5_LUHS122_	25.01	17.48	57.52	3.38	54.14	17.48

LUHS29—treated with *Pediococcus acidilactici* LUHS29; LUHS245—treated with *Liquorilactobacillus uvarum* LUHS245; LUHS122—treated with *Lactiplantibacillus plantarum* LUHS122.

Wheat kernel grains contain, on average, 3.1% oil on a dry basis (Lafiandra et al., 2012). The majority of lipids are located in the germ (66%), and the average lipid content in the bran and endosperm is 15 and 20% (Narducci et al., 2019). The wheat cereal FA profile mostly consists of unsaturated ones, of which linoleic and linolenic acids are the most important (Narducci et al., 2019), because they are involved in physiological processes of blood lipid metabolism (Lafiandra et al., 2012). However, the lipid content and FA profile are related to many factors (Lafiandra et al., 2012), such as cold weather (Nejadsadeghi et al., 2015), as well as biotic and abiotic stresses (Upchurch, 2008). Also, the differences in SFA and UFA within the same cereal variety could be related to various biotic and abiotic stresses (Upchurch, 2008; Nejadsadeghi et al., 2015). This study showed that the technological treatment is also important and could modulate the FA profile of WCW. It was established that the LAB strain used for fermentation was significant factor on C14:0, C16:1, C18:0 and C20:1 content in WCW samples (*p* = 0.024, *p* = 0.0001, *p* = 0.0001, and *p* = 0.001, respectively).

It was reported, that the major component of wheat fat is linoleic acid (52.16% for durum wheat, and 59.10% for common wheat, as well as the content of UFA in triticale is, on average, 79.51%, in durum wheat –78.14%, and common wheat –77.97% (Kan, 2015).

Also, biotechnological solutions, including fermentation, have been used to enrich cereals with PUFA (Certik and Adamechova, 2009). Fat content in cereal grains is, on average, 3.6%, while linoleic acid is the major FA. Also, during the fermentation, various changes can be obtained, including lipids bioconversion. It was reported, that LAB can perform FA isomerization, hydration, dehydration, and saturation in fermentable substrate (Ogawa et al., 2005).

The metabolism of LAB in fermented cereal can favor lipid oxidation during fermentation, or exert strong antioxidative effects (Gänzle, 2014). Wheat lipoxygenase preferably forms 9 hydroperoxy linoleic acid (Belitz et al., 2004). Hydroperoxy linoleic acid is alternatively reduced to hydroxy-linoleic acid with concomitant oxidation of other flour constituents. In presence of cysteine, peroxides are converted to the corresponding hydroxy-fatty acids (Shahzadi, 2012). Heterofermentative LAB decrease the oxidationereduction potential of fermented cereal, and specifically accumulate glutathione or related low-molecular weight thiol compounds (Jänsch et al., 2007; Capuani et al., 2012). Thiol accumulation through heterofermentative metabolism is linked to the generation of reducing equivalents in the pentose phosphate pathway (Jänsch et al., 2007), providing abundant reducing power to convert lipid peroxides to hydroxides (Gänzle, 2014). Lactobacilli hydrate oleic, linoleic, and linoleic acids to hydroxyl FA. Linoleic acid is converted to 13-hydroxy-9-octadecenoic acid or the antifungal 10-hydroxy-12-octadecenoic acid (Ogawa et al., 2001; Shahzadi, 2012). The reaction is catalyzed by a FA hydratase (Volkov et al., 2010; Yang et al., 2013). However, the data about FA content in WCW (non-treated and fermented with *Liquorilactobacillus uvarum* (strain LUHS245), *Pediococcus acidilactici* [strain LUHS29) and *Lactiplantibacillus plantarum* (strain LUHS122)] is scarce. From this point of view, the results of this study can be valuable for data basis about WCW FA profile, as well as it changes during fermentation.

### Volatile compound profile of non-treated and fermented wheat cereal wholemeal samples

The VC with concentrations above 5% in at least one WCW sample are shown in Table 4. Acetic acid (responsible for the pungent, sour, overripe, fruit odor) was found in two fermented samples (DS8472-5 LUHS29 and DS8472-5 LUHS122). Acetoin (responsible for a buttery odor and flavor) was only detected in non-fermented WCW (6.65% of the total VC content). The highest content of 2,3-butanediol was found in non-fermented WCW; nonetheless, after fermentation, its content was reduced in DS8472-5 LUHS245 samples by 4.4 times and in DS8472-5 LUHS122 by 27.7 times; this VC was not found in DS8472-5 LUHS29 samples. The compound 2,3-butanediol imparts a fruity, creamy and buttery odor. Ethyl lactate (which gives an odor described as sweet, fruity, acidic and ethereal with a brown nuance) was only detected in DS8472-5 LUHS29 samples. The 1-hexanol (which imparts a pungent, ethereal, fuel oil, fruity and alcoholic, sweet odor with a green top note) was found in all the analyzed samples, and the highest content was detected in non-fermented and DS8472-5 LUHS245 samples (on average, 10.01% of the total VC content). The 1-L-lactic acid (odorless acidic) was found in DS8472-5 LUHS245 samples. Half of the entire VC profile in DS8472-5 LUHS122 samples consisted of 4-methylpentanoic acid, which has an odor described as that of pungent cheese. The highest content of 2-heptenal (intense green, fatty, oily, odor with fruity overtones) was identified in the VC profile of DS8472-5 LUHS245. 1-Octen-3-ol (earthy, green, oily, vegetative and fungal odor) was found in non-fermented samples and those fermented with *P. acidilactici* LUHS29 and *Liq. uvarum* LUHS245. Hexanoic acid (sour, fatty, sweaty, and cheesy odor) was only observed in fermented samples, and the highest content was found in DS8472-5 LUHS29 samples. The 2-pentylfuran (fruity, green, earthy beany odor with vegetable-like nuances) was found in non-fermented samples and in those fermented with *P. acidilactici* LUHS29 and *Liq. uvarum* LUHS245; however, in DS8472-5 LUHS122 samples, such VC was not detected. Hexanoic acid ethyl ester, 3-ethyl-2-methyl-1,3-hexadiene, benzeneacetaldehyde, and 4-vinylguaiacol were only detected in fermented samples. The odor of hexanoic acid ethyl ester is described as sweet, fruity, pineapple, waxy, fatty, and estery with a green banana nuance, and that of benzeneacetaldehyde is described as honey, floral rose, sweet, powdery, fermented, and chocolate with a slight earthy nuance. The odor of 4-vinylguaiacol has sweet, musty and meaty nuances. In contrast to the abovementioned VC, 4-methyl-1-(pent-4-en-1-yl)-2,3-diazabicyclo[2.2.1] hept-2-ene was only found in non-fermented WCW. Octanoic acid was detected in all the tested WCW, with the highest content in DS8472-5 LUHS29. The odor of octanoic acid is described as fatty, waxy, rancid, oily, vegetable, and cheesy. Oct-(2E)-enal showed different tendencies, as it was found in non-fermented samples and in DS8472-5 LUHS245 and DS8472-5 LUHS122 WCW. The odor of oct-(2E)-enal is described as fresh, cucumber, fatty, green, herbal, banana, waxy and leafy green.

**TABLE 4:** Volatile compounds (VC) present in at least one wheat cereal wholemeal (WCW) (DS8472-5) sample at > 5%.

RT (min)	Volatile compound	DS8472-5	DS8472-5_LUHS29_	DS8472-5_LUHS245_	DS8472-5_LUHS122_
2.34	Acetic acid	nd	23.3 ± 2.2^b^	nd	6.36 ± 0.32^a^
3.75	Acetoin	6.65 ± 0.59	nd	nd	nd
5.50	2,3-Butanediol	53.8 ± 4.8^c^	nd	12.3 ± 1.2^b^	1.94 ± 0.09^a^
6.10	Ethyl lactate	nd	5.99 ± 0.57	nd	nd
7.57	1-Hexanol	9.89 ± 0.89^c^	7.78 ± 0.74^b^	10.13 ± 1.01^c^	0.321 ± 0.016^a^
8.04	L-Lactic acid	nd	nd	9.33 ± 0.93	nd
9.74	4-methylpentanoic acid	nd	nd	nd	54.8 ± 4.7
9.87	2-Heptenal	0.910 ± 0.082^b^	0.640 ± 0.064a	2.69 ± 0.27^c^	nd
10.4	1-Octen-3-ol	0.445 ± 0.029^a^	5.68 ± 0.34^c^	3.09 ± 0.17^b^	nd
10.5	Hexanoic acid	nd	9.07 ± 0.86^c^	5.04 ± 0.50^b^	0.753 ± 0.038^a^
10.7	2-pentylfuran	1.26 ± 0.11^a^	4.76 ± 0.45^b^	4.74 ± 0.47^b^	nd
11.0	Hexanoic acid ethyl ester	nd	5.07 ± 0.48^b^	3.45 ± 0.35^a^	22.4 ± 1.1^c^
11.8	3-ethyl-2-methyl-1,3-hexadiene	nd	3.53 ± 0.34^b^	9.83 ± 0.98^c^	1.58 ± 0.08^a^
12.1	Benzeneacetaldehyde	nd	3.22 ± 0.31^c^	2.51 ± 0.25^b^	1.18 ± 0.06^a^
12.4	Oct-(2E)-enal	1.61 ± 0.14^b^	nd	6.37 ± 0.64^c^	0.664 ± 0.033^a^
15.1	Octanoic acid	0.892 ± 0.058^b^	5.23 ± 0.31^d^	2.26 ± 0.12^c^	0.651 ± 0.049^a^
18.38	4-vinylguaiacol	nd	3.06 ± 0.29^b^	6.66 ± 0.66^c^	1.67 ± 0.08^a^
18.39	4-methyl-1-(pent-4-en-1-yl)-2,3-diazabicyclo[2.2.1] hept-2-ene	8.07 ± 0.52	nd	nd	nd

LUHS29—treated with *Pediococcus acidilactici* LUHS29; LUHS245—treated with *Liquorilactobacillus uvarum* LUHS245; LUHS122—treated with *Lactiplantibacillus plantarum* LUHS245; RT—retention time, in minutes. Mean values (*n* = 3) ± standard error (SE). ^a–c^Means with different letters in the same column are significantly different (*p* ≤ 0.05). nd, not detected.

The VC concentrations that were > 1 and < 5% in at least one WCW sample and the VC of which the content in WCW was < 1% of the total VC are given in Supplementary Table 1. Conversely, the VC of which the content in WCW was < 1% of the total VC content are given in Supplementary Table 2.

It was reported, that between the yellow and pigmented wheat kernels VC compounds are qualitative differences, however, the main VC are aldehydes and alcohols, and to a lesser extent, terpenes, and benzene derivatives (Germinara et al., 2019). Wheat variety is significant factor on VC profile, and, modern wheat varieties are characterized by higher content of terpenes, pyrazines, and straight-chained aldehydes (Starr et al., 2015). Seitz reported, that acohols were most abundant in wheat, followed in order by aldehydes, alkanes, alkylbenzenes, ketones, methyl esters, naphthalenes, terpenes, and other miscellaneous compounds (Seitz, 1995). Also, it was published, that wheat bran VC profile consist mainly from alcohols, aldehydes, ketones, carboxylic acids, furan derivatives, and esters, and fermented wheat bran showed completely different aroma notes in comparison with unfermented bran (Spaggiari et al., 2020). However, fermentation could change VC profile in relation with used technological strain characteristics, fermentation conditions (temperature, water content, etc.). Results of the sourdoughs fermentation at 28 and 35 °C by nine different combinations between *Lactobacillus amylovorus*, *Lactobacillus brevis*, *Lactobacillus plantarum*, *Lactobacillus reuteri*, and *Pediococcuspentosaceus* showed, that VC profile was influenced by temperature and starter culture, and, at 35 °C more aldehydes, esters and fewer alcohols were found (Siepmann et al., 2019). The impact of *Lactobacillus delbrueckii* ssp. *bulgaricus* MI, *L. rossiae* GL14, and *L. acidophilus* DSM 20079 on whole-grain wheat and sourdough bread VC profile was studied (Cizeikiene et al., 2020). The authors found, that the compositions of VC in sourdough and whole-grain wheat bread depended on the LAB strain used, and in bread with *L. acidophilus*, 3-octen-2-ol and n-hexadecane were found, whereas those compounds were not found in other bread samples or in sourdough; tetrahydrofurfuryl acetate was found in with *L. bulgaricus*MI fermented sourdough; N-pentadecane—in bread prepared with *L. rossiae* sourdough.

Finally, the results of VC profile formation in blue wheat variety DS8472-5 wholemeal samples showed, that the used LAB strains are suitable to degrade fermentable substrate and to form broad spectrum of VC.

### Microbial profiles of fermented and non-fermented wheat cereal wholemeal samples

**FIGURE 6:**
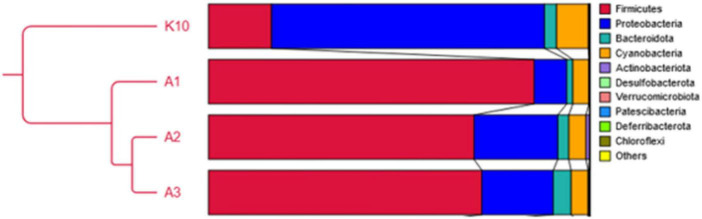
Relative abundance of bacteria at a phylum level. K10, non-fermented sample; A1, sample fermented by P. *acidilactici*; A2, fermented by *L. plantarum*; A3, fermented by *L. uvarum*.

**FIGURE 7:**
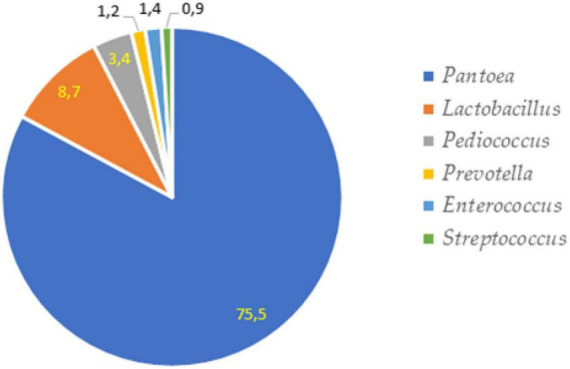
The most prevalent bacterial genera in non-fermented wheat cereal wholemeal (WCW) (DS8472-5) samples.

**FIGURE 8:**
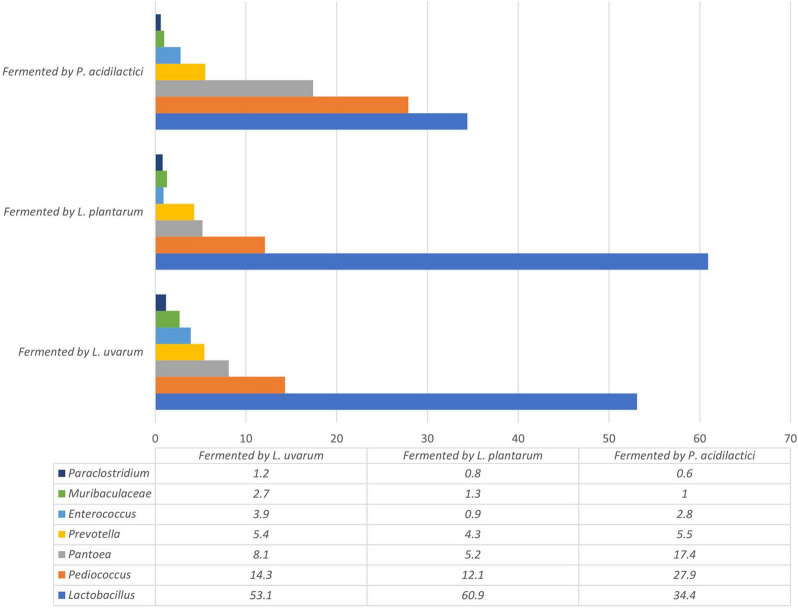
The most prevalent bacterial genera, the prevalence of which was at least 1% of all bacterial observations in any group, in fermented wheat cereal wholemeal (WCW) (DS8472-5) samples.

The most prevalent phylum in non-fermented samples were Proteobacteria which consisted of more than 75% from all microbiota. The second most prevalent phylus was Firmucutes. The main phyla in-fermented samples included Firmucutes (72.8–85.8%), Proteobacteria (6.0–18.1%) and Bacteroidota (7.1–8.8%). The relative abundance of bacteria at a phylum level is presented in Figure 6. The bacterial profiles, at the genus level, in non-fermented and fermented WCW samples are presented in Figures 7, 8, respectively. In total, 81,811 reads of bacterial DNA in non-fermented WCW were obtained after next-generation sequencing. Non-fermented WCW of *Triticum aestivum* mostly contained *Pantoea* spp., the prevalence of which was at least 75.5% of the total bacterial viable counts. Other predominant genera included *Lactobacillus* (8.7%), *Pediococcus* (3.4%), *Enterococcus* (1.4%), *Prevotella* (1.2%) and *Streptococcus* (0.9%) (Figure 7). Figure 8 demonstrates the bacterial composition of fermented WCW samples.

Fermentation significantly reduced the number of *Pantoea*, which was the most prevalent bacterial genus in non-fermented WCW. *Pantoea* is a gram-negative aerobic bacillus from the family Enterobacteriaceae. All species of the genus *Pantoea* can be isolated from feculent material, plants and soil (Andersson et al., 1999), where they can be either pathogens or commensals (Monier and Lindow, 2005). Other genera, such as *Enterococcus*, *Prevotella* and *Paraclostridium*, and the bacterial family Muribaculaceae, were also found in fermented WCW in large percentages (0.6–5.5%). The most prevalent bacteria in all fermented WCW were *Lactobacillus*, followed by *Pediococcus*, irrespective of the bacterial cultures used for fermentation. This means that *Lactobacillus* spp. has better growth potential rather to other microbiota. Besides medium composition, the growth of *Lactobacillus* spp. can be influenced by a set of various conditions including temperature, pH, oxygen concentration and water activity (Davoodi et al., 2016; Dalcanton et al., 2018). The optimum temperature and pH conditions for lactobacilli growth are 30–40°C and pH varying between 4.5 and 6.5, some strains can grow in even lower pH (König and Berkelmann-Löhnertz, 2017). Therefore the condition used was optimal for the growth of this group of bacteria. As *Lactobacillus* was detected in higher amounts than *Pediococcus* itself in WCW fermented by *P. acidilactici* (and high numbers of *Pediococcus* were also detected in samples fermented by *Lactobacillus* spp.), it may be stated that LAB, which are naturally prevalent in WCW, also participate actively in the fermentation process. This should be kept in mind in the case of industrial bacterial cultures intended for use in fermentation processes on substrates of plant origin, where the proliferation of the prevalent microbiota in raw samples cannot be ignored. There are data that microorganisms in fermented food and feed can survive, positively modify microbiota within the gut and to have positive influence on immune status of animals (Chan et al., 2021; Peng et al., 2022). It should also be mentioned that although fermentation reduces the undesirable components of the natural microbiota, it does not eliminate them. This fact was also proved by other authors (Leeuwendaal et al., 2022). Therefore, fermented products may contain different bacterial genera from those presented in a raw product before fermentation. However, as DNA-based methods (next-generation sequencing in this case) used for the detection of microorganisms did not provide any information on whether the microorganisms were viable or not, culture-based methods can be further used to ensure the viability of bacteria. On the other hand, culture-based methods may be useful only if the microorganisms are cultivable (Kumar and Ghosh, 2019) and, therefore, the viability of only a limited genera can be determined. In this study the methods used do not provide information on the viability of microorganisms in fermented products as well as there is no information whether the most prevalent bacterial strains of LAB in fermented products were the same as the starter strains. Further studies using bacterial identification at the strain level, such as RAPD-PCR or REP-PCR should be performed.

## Conclusion

All the tested LAB were suitable for DS8472-5 WCW fermentation, and the lowest pH and the highest TTA and LAB counts (3.94, 5.80°N, and 8.92 log_10_ CFU/g, respectively) could be obtained by using the strain *Lp. plantarum* LUHS122 for fermentation. Sourdough fermentation influenced the color of the WCW and increased the FAA content (except that of arginine, glutamine and serine in DS8472-5 LUHS29; glutamine, serine, aspartic and glutamic acids and alanine in DS8472-5 LUHS245 and glutamine, aspartic and glutamic acids and histidine in DS8472-5 LUHS122. Fermentation with LUHS122 increased the GABA concentration in WCW by a factor of 3.31. Sourdough fermentation also led to the formation of spermidine and cadaverine (in DS8472-5 LUHS122) and PHE was found in all WCW samples (fermented and non-fermented ones). The LAB strain used for fermentation was a significant factor in the concentrations of Na, K, and Cr in WCW. Fermented samples showed positively higher ω-3 and ω-6 contents than non-fermented ones but a lower ω-9 content. Fermentation also increases the number and variety of VC in WCW. Further research is needed to evaluate the influence of prepared sourdoughs and their different constituents on bread quality.

## Data availability statement

The original contributions presented in this study are included in the article/Supplementary material, further inquiries can be directed to the corresponding author.

## Author contributions

EB and VR: conceptualization and supervision. VS, EZ, DK, EM, MR, VB, AB, FO, ZL, and VR: methodology and investigation. VS and EZ: software and visualization. VS, EZ, DK, and EM: validation. VS, EZ, DK, AB, and EM: formal analysis. EB, VB, and VR: resources. VS, EZ, DK, EM, MR, VB, AB, FO, JR, ZL, and VR: data curation. EB, VS, EZ, DK, and VB: writing—original draft preparation. EB, VR, ZL, VS, EZ, FO, JR, and VB: writing—review and editing. EB: project administration. All authors read and agreed to the published version of the manuscript.
