# Supplementary material for: Changes in the physicochemical parameters and microbial community of a new cultivar blue wheat cereal wholemeal during sourdough production
Source: Front Microbiol. 2022 Dec 8;13:1031273. doi: 10.3389/fmicb.2022.1031273 (PMC9773212; doi:10.3389/fmicb.2022.1031273)
Supplement: Supplementary file 1 [file Data_Sheet_1.docx]

Supplementary Material

# S1. Description of the field trials

A set of field trials were conducted during the biennium 2020–2021 at the experimental base of the LAMMC in Akademija, Kėdainiai distr., Lithuania (55°39′ N 23°57′ E) (**Figure S1**).


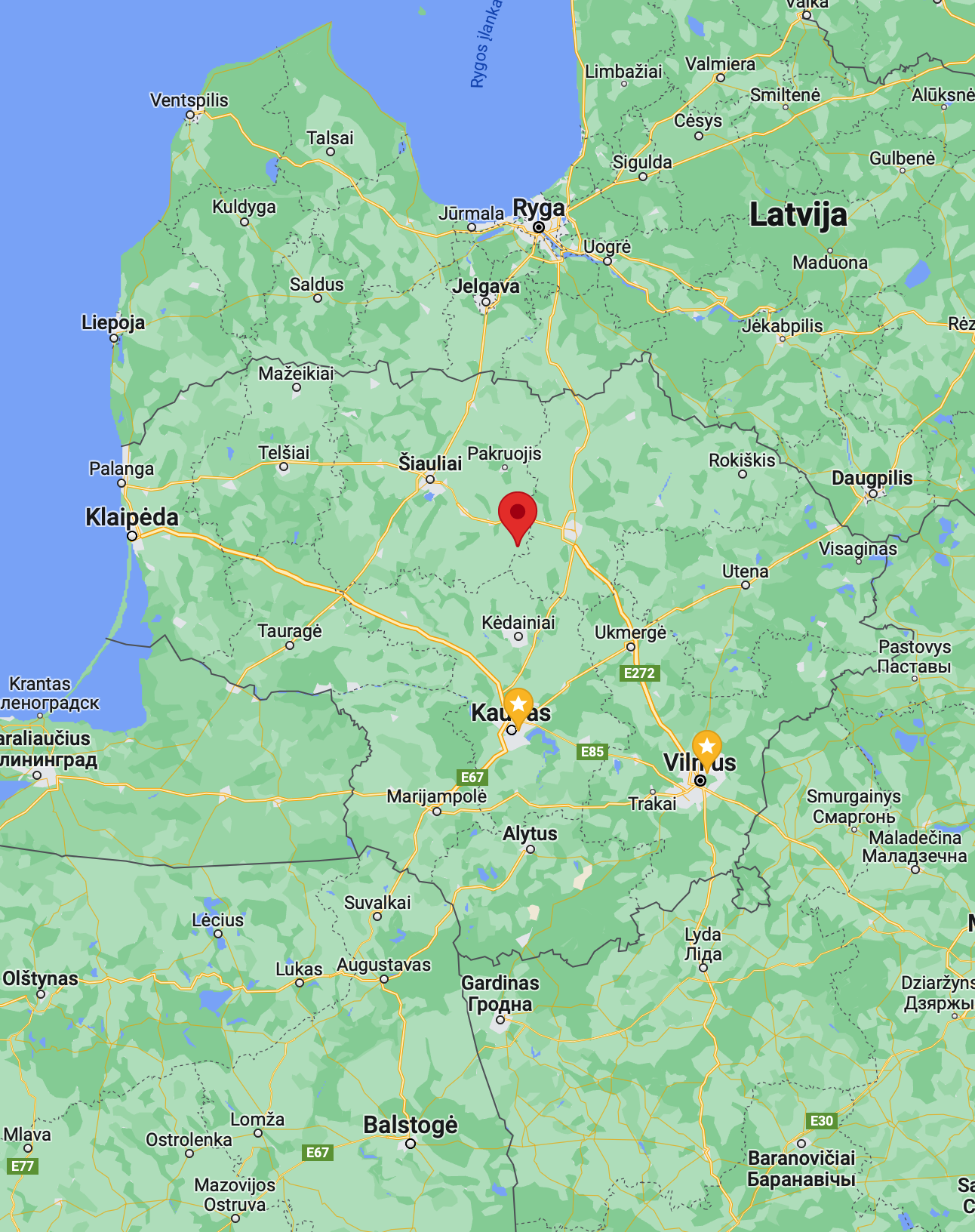


**Figure S1**. Place of the experimental base of the LAMMC in Akademija, Kėdainiai distr., Lithuania.

Field experiment designed in 4 replications (plot size 5×1.6 m), each replication was grown in a separate block, where the field plots were arranged randomized.

The trials were conducted under sustainable growing technology.

The soil was light loam Endocalcari-Epihypogleyic-Cambisol. Topsoil (0–30 cm) pH is low acid (5.5), close to low in humus (1.6 %); high in available phosphorus (251 mg/kg P_2_O_5_) and moderate in available potassium (175 mg/kg K_2_O).

Winter wheat was sown with treated seeds at the seed rate of 4.5 million/ha in the beginning 10^th^ of September (2020) after the black fallow.

In every year, complex mineral fertilizers (N_15_P_50_K_100_) were applied in the whole experimental field before sowing. Nitrogen fertilizers (ammonium nitrate) were applied after resumption of spring vegetation (at 8^th^ of April 2021) and when plants reached stem elongation stage (at 4^th^ of May, 2021). The rate of N_100+30_ was used.

Weeds were controlled by the recommended herbicides in the autumn and spring.

Yield was harvested on 21^st^ of July. The elevation of experimental area is 82 meters above sea level, belongs to the mid-latitude climate zone in the southwestern sub-region of the Atlantic continental forest area.

According to the data of local Dotnuva Meteorological Station (55°23'49.0"N 23°51'55.0"E), the climatic conditions were characterized by the long-term (1924–2021) average annual temperature of 6.5 °C and precipitation of 570 mm. Both experiment growing seasons, especially of spring - summer period, were warmer (2.3 °C), as well 2020-2021 growing season – was dryer than the long-term average.

The Autumn of 2020 was warmer and dryer than usually. The winters were quite hard, thick snow cover protected plant from cold damage, but provoked intense development of snow mold.

The Spring meteorological conditions were variable – March was warmer and dryer, April colder and dryer and May was extremely cold and wet as precipitations was twice as much.

The drought period was recorded from middle June to end of July. June and July were warmer by 3.1 and 4.7 °C, respectively. The first part of July was warmer by 6.5 °C, this affected the rapid maturation of plants which formed small grains.

# S2. Chemical analysis of free amino acids (FAA), fatty acids (FA) and volatile compounds (VC)

## S2.1. Analysis of free amino acids (FAA) in wheat cereal wholemeal (WCW) samples

Sample preparation and dansylation were performed according to the method of Hua-Lin Cai et al. [28], with some modifications. The homogenized sample (~100 mg) was weighed into a 1.5 mL tube, and analytes were extracted with 1 mL of aqueous 0.1 M HCl solution by shaking for 1 h. The resultant mixture was centrifuged at 12,000 rpm for 5 minutes. For derivatization, 50 µL of the resultant supernatant was mixed with 100 µL of 100 mg/L diaminoheptane (as an internal standard) and diluted to 500 µL with 0.1 M HCl solution. The resultant mixture was alkalized by addition of 40 µL of 2 M NaOH and 70 µL of the saturated NaHCO_3_ solution. Derivatization was performed by adding 1 mL of 10 mg/mL dansyl chloride solution in acetonitrile and incubating the resulting mixture at 60 ℃ for 30 minutes. The reaction mixture was quenched using 50 µL of 25% ammonia solution and filtered through a 0.22 µm membrane filter into the auto-sampler vial. The concentration of analytes was determined using The Varian ProStar HPLC system ([Varian Corp., Palo Alto, California, USA]: two ProStar 210 pumps and a ProStar 410 auto-sampler) and Thermo Scientific LCQ Fleet Ion trap mass detector. For analyte detection, the mass spectrometer was operated in positive-ionization single-ion monitoring mode for specific ions corresponding to derivatized analytes. The analyte concentration was determined from a calibration curve, which was obtained by derivatizing the analytes at different concentrations. For the separation of derivatives, a Discovery^®^ HS C18 column (150 × 4.6 mm, 5 µm; SupelcoTM Analytical, Bellefonte, Pennsylvania, USA) was used. Mobile phase A was 0.1% formic acid in 5% aqueous acetonitrile, and phase B was 0.1% in acetonitrile. A flow-rate of 0.3 mL/min was used for the analysis. The injection volume was 10 µL. The analytical gradient was as follows: 0 to 10 min (linear gradient) 15 to 60% B, 10 to 40 min (linear gradient) 60 to 95 % B, 40 to 48 min 95 B, followed by re-equilibration for 10 minutes with 15% B (increased to 0.6 mL/min flowrate). The limit of quantification (according to the lowest concentration used for calibration) was 0.02 µmol/g.

## S2.2. Analysis of wheat cereal wholemeal (WCW) fatty acid (FA) profiles

The fatty acid (FA) composition of WCW samples was determined using a GCMS-QP2010 (Shimadzu, Japan) gas chromatograph with a mass spectrometer. The fatty acid methyl ester (FAME) concentration was determined using a calibration curve, and the results were expressed as a percentage of the total FA in the sample.

The sample was prepared by homogenizing 1 g of WCW sample in 5 mL of 30% (w/v) NaCl solution. Next, 5 mL of hexane were added. The mixture was shaken on a laboratory shaker for 1 h and then centrifuged at 4000 rpm. Afterwards, 4 mL of the hexane extract were reacted with 300 µL of methylation reagent (2 mol/L of KOH in methanol) by vortexing and shaking using a laboratory shaker for 1 h. The mixture was centrifuged at 4000 rpm, and the upper layer was filtered using a 0.22 µm membrane syringe filter and used for the analysis. A capillary Stabilwax-MS column (30 m × 0.25 mm ID × 0.25 µm film thickness was used, and the mass spectrometer was operated in full scan mode. The analyte was injected in split mode at a 1:60 split ratio. The following parameters were used: MS ion source temperature: 240 °C, MS interface temperature 240 °C, helium (carrier gas) flow: 0.90 mL/min, injector: 240 °C, oven temperature 50 °C (4 min), 10 °C/min to 110 °C (1 min), 15 °C/min to 160 °C (2 min), 2.5 °C/min to 195 °C (1 min), 2 °C/min to 230 °C (1 min), 2 °C/min to 240 °C (12 min).

## S2.3. Evaluation of the volatile compound (VC) profiles of non-treated and fermented wheat cereal wholemeal (WCW) samples

The VCs of the WCW samples were analyzed by gas chromatography-mass spectrometry (GC-MS). A solid phase microextraction (SPME) device with Stableflex™ fibre coated with a 50 µm PDMS-DVB-Carboxen™ layer (Supelco, USA) was used for the analysis.

A WCW was weighed and blended with aqueous sodium chloride solution (30% w/v) in a ratio of 1 g of WCW to 3 mL of NaCl solution. For headspace extraction, 8 g of prepared sample was transferred to a 20 mL extraction vial, sealed with a polytetrafluoroethylene septum, and placed in a thermostated oven at 60 °C for 15 min before exposing the fiber in the headspace. The fiber was exposed to the headspace of the vial for 10 min and desorbed in an injector liner for 2 min (splitless injection mode). Prepared samples were analyzed with a GCMS-QP2010 (Shimadzu, Japan) gas chromatograph and mass spectrometer. The following conditions were used for the analysis: injector temperature 250 °C, ion source temperature 220 °C and interface temperature 260 °C. Helium was used as the carrier gas at a flow-rate of 0.95 mL/min. A Stabilwax-DA capillary column (0.25 mm ID, 0.25 μm film thickness, 30 m length [Restek, USA]) was used for the analysis. The temperature gradient was programmed from a start at 40 °C (3 min hold) to 220 °C (6 °C/min) up to 250 °C (10 °C/min) (6 min hold). The VC were identified according to mass spectrum libraries (NIST11, NIST11S and FFNSC2).

# S3. Results of volatile compounds

## The VC concentrations that were >1% and <5% in at least one WCW sample and the VC of which the content in WCW was <1% of the total VC content are given in Tables S1 and S2, respectively.

## S3.1. Volatile compounds (VC) of which the content in wheat cereal wholemeal (WCW) sample was >1% and <5% of the total VC

The VC of which the content in WCW was >1% and <5% of the total VC content is presented in **Table S2**. Heptanoic acid, phenethyl alcohol, 4-ethylphenol and 4-ethyl-3-nonen-5-yne were found only in fermented samples. The odor of heptanoic acid is sour, fatty, sweaty and cheesy; that of phenethyl alcohol is floral, rose, dried rose, flower and rose water and that of 4-ethylphenol is described as smoky, phenolic, creosote and savory. Nonanal, ethyl octanoate, nonanoic acid, dihydro-5-pentyl-2(3H)-furanone and dodecanoic acid were present in all non-fermented and fermented samples. The odor of nonanal is aldehydic, citrus and fatty; that of ethyl octanoate is described as fruity, wine, waxy, sweet, apricot, banana, brandy and pear; the odor of nonanoic acid is waxy, dirty and cheesy, with a cultured dairy nuance and the aroma of dodecanoic acid is sour and fatty. The 4-methylhexanoic acid and ethylhydrocinnamate were found only in DS8472-5 LUHS122 samples. The aroma of 4-methylhexanoic acid is sour and cheesy, and that of ethylhydrocinnamate is described as hyacinth, rose, honey, fruity and rum. The 2,5-dimethylpyrazine (nutty, peanut, musty, earthy, powdery and slightly roasted with a cocoa powder nuance odor) was formed in DS8472-5 LUHS29 and DS8472-5 LUHS122 samples. The 1-hHeptanol and 2-nonanone were present in non-fermented samples and in those fermented with LUHS29 and LUHS245. 1-Heptanol possesses a musty, pungent and leafy green odor with vegetative and fruity nuances of apple and banana, and the odor of 2-nonanone is described as fresh, sweet, green, weedy, earthy and herbal. 4-ethylguaiacol (spicy and clove-like odor with medicinal, woody and sweet vanilla nuances) was formed in WCW samples fermented with *Liq. uvarum* LUHS245 and in those fermented with *Lp. plantarum* LUHS122. The highest content of (E,E)-2,4-decadienal was found in non-fermented samples; however, after fermentation with *P. acidilactici* LUHS29 and *Lp. plantarum* LUHS122, its content was reduced (on average, by factors of 3.8 and 14.9, respectively), and this VC was not detected in DS8472-5 LUHS245. The (E,E)-2,4-decadienal odor is described as fatty, chicken, fried, citrus, chicken fat, coriander and brothy.

**Table S1**. Volatile compounds (VC) that were present at >1 and <5% in at least one wheat cereal wholemeal (WCW) (DS8472-5) sample.

| **RT (min)** | **Volatile compound** | **DS8472-5** | **DS8472-5_LUHS29_** | **DS8472-5_LUHS245_** | **DS8472-5_LUHS122_** |
| --- | --- | --- | --- | --- | --- |
| 8.71 | 2,5-dimethylpyrazine | nd | 1.27±0.08^b^ | nd | 0.434±0.043^a^ |
| 10.2 | 1-Heptanol | 0.549±0.036^a^ | 1.83±0.11^c^ | 0.766±0.042^b^ | nd |
| 12.3 | 4-methylhexanoic acid | nd | nd | nd | 1.44±0.14 |
| 12.8 | Heptanoic acid | nd | 1.52±0.09^c^ | 0.522±0.029^b^ | 0.171±0.017^a^ |
| 13.3 | 2-Nonanone | 0.381±0.025^a^ | 0.573±0.034^b^ | 1.26±0.07^c^ | nd |
| 13.6 | Nonanal | 1.50±0.10^d^ | 0.217±0.013^b^ | 0.342±0.019^c^ | 0.103±0.01^a^ |
| 13.8 | Phenethyl alcohol | nd | 1.14±0.07^a^ | 1.09±0.06^a^ | 0.265±0.027^b^ |
| 15.0 | 4-ethylphenol | nd | 0.939±0.055^b^ | 1.14±0.06^c^ | 0.091±0.090^a^ |
| 15.7 | Ethyl octanoate | 1.10±0.07^c^ | 1.37±0.08^d^ | 0.876±0.048^b^ | 0.329±0.031^a^ |
| 17.2 | Nonanoic acid | 1.51±0.09^b^ | 4.33±0.26^d^ | 2.28±0.13^c^ | 0.262±0.060^a^ |
| 17.6 | 4-ethylguaiacol | nd | nd | 1.02±0.10^b^ | 0.216±0.011^a^ |
| 17.9 | (E,E)-2,4-Decadienal | 3.54±0.23^c^ | 0.931±0.056^b^ | nd | 0.237±0.024^a^ |
| 18.4 | 4-ethyl-3-Nonen-5-yne | nd | 0.406±0.024^b^ | 3.03±0.17^c^ | 0.209±0.022^a^ |
| 19.0 | Ethylhydrocinnamate | nd | nd | nd | 1.06±0.11 |
| 19.4 | dihydro-5-pentyl-2(3H)-Furanone | 0.528±0.034^b^ | 1.05±0.06^d^ | 0.931±0.051^c^ | 0.223±0.021^a^ |
| 23.0 | Dodecanoic acid | 0.113±0.007^b^ | 2.06±0.12^d^ | 0.543±0.029^c^ | 0.046±0.005^a^ |
| LUHS29 – treated with *Pediococcus acidilactici* LUHS29; LUHS245 – treated with *Liquorilactobacillus uvarum* LUHS245; LUHS122 – treated with *Lactoplantibacillus plantarum* LUHS122; RT – retention time, in minutes; Mean values (n = 3) ± standard error (SE). ^a-c^ Means with different letters in column are significantly different (p ≤ 0.05). nd – not detected. | | | | | |

## S3.2. Volatile compounds (VC) of which the content in wheat cereal wholemeal (WCW) was <1% of the total VC content

The VC of which the content in WCW was <1% of the total VC content are shown in **Table S2**. (E)-2-Octen-1-ol (green, citrus, vegetable and fatty odour), (E)-2-nonenal (green, cucumber, aldehydic and fatty with a citrus nuance odour), dodecane, (E,E)-2,4-nonadienal (fatty, green and cucumber odor), tetradecane (mild and waxy odor) and pentadecane (waxy odour) were found in all (non-fermented and fermented) WCW samples. Undecane, dec-(2E)-enal and deca-(2E,4E)-dien-1-ol were only found in non-fermented WCW samples. The odor or dec-(2E)-enal is described as waxy, fatty, earthy, green, cilantro, mushroom, aldehydic, fried chicken, fat and tallow, and that of deca-(2E,4E)-dien-1-ol odor is described as fatty and waxy, like chicken white meat and turkey, with a slight melon fruity and dairy nuance. The 2-butyltetrahydrofuran, 2-(ethylthio)propanoic acid ethyl ester, sorbic acid vinyl ester, benzothiazole and benzo-2,3-pyrrole were formed only in DS8472-5 LUHS122 samples. The odor of benzothiazole is described as sulfureous, rubbery, vegetative, cooked, brown, nutty, coffee-like and meaty, and that of benzo-2,3-pyrrole odor is sweet, musty, nutty and tea-like. Benzeneacetic acid ethyl ester (sweet, floral, honey, rose, balsam and cocoa odor) and trans-4,5-epoxy-(E)-2-decenal (citrus, metallic, green and aldehydic odor) were found in WCW fermented with *Liq. uvarum* LUHS245 and *Lp. plantarum* LUHS122. Acetic acid hexyl ester (green, fruity, sweet, fatty, fresh, apple and pear odor), 4a,5-dimethylhexahydro-4H-1,3-benzodioxin-4-one, 2-undecanone and cis-4,5-epoxy-(E)-2-decenal were formed only in WCW samples fermented with LUHS245. Tridecane, n-decanoic acid and 2,4,7,9-tetramethyl-5-decyn-4,7-diol were found in non-fermented samples and those fermented with *P. acidilactici* LUHS29 and *Liq. uvarum* LUHS245. The odor of n-d acid is described as unpleasant, rancid, sour, fatty and citrus. Decanal and 2,6,10-trimethyldodecane were present in non-fermented samples and those fermented with *Liq. uvarum* LUHS245 and *Lp. plantarum* LUHS122 strains WCW samples. The 5,5,6-trimethylhept-3-en-2-one and 4,6-dimethyldodecane were formed in non-fermented WCW samples and in those fermented with *P. acidilactici* LUHS29. Heptanoic acid ethyl ester (fruity, pineapple, cognac, rum and wine odor) was found in samples fermented with LUHS29 and LUHS122, and 2,4-bis(1,1-dimethylethyl)phenol was present in WCW fermented with LUHS29 and LUHS245 . The 1-octanol and benzoic acid ethyl ester were found in non-fermented samples, and 1-octanol was established in WCW fermented with *Liq. uvarum* LUHS245 and benzoic acid ethyl ester in WCW fermented with *Lp. plantarum* LUHS122. The odor or 1-Octanol is described as waxy, green, citrus, aldehydic, and floral with a sweet, fatty, coconut nuance, and that of benzoic acid ethyl ester is fruity, dry, musty, sweet and wintergreen.

**Table S2**. Volatile compounds (VC) of which the content in wheat cereal wholemeal (WCW) (DS8472-5) samples was <1%.

| **RT (min)** | **Volatile compound** | **DS8472-5** | **DS8472-5_LUHS29_** | **DS8472-5 _LUHS245_** | **DS8472-5_LUHS122_** |
| --- | --- | --- | --- | --- | --- |
| 11.3 | Acetic acid hexyl ester | nd | 0.351±0.028 | nd | nd |
| 12.7 | (E)-2-Octen-1-ol | 0.353±0.032^b^ | 0.574±0.046^c^ | 0.578±0.041^c^ | 0.192±0.012^a^ |
| 12.7 | 1-Octanol | 0.608±0.055^a^ | nd | 0.638±0.044^a^ | nd |
| 13.3 | 2-butyltetrahydrofuran | nd | nd | nd | 0.277±0.017 |
| 13.4 | Heptanoic acid ethyl ester | nd | 0.184±0.015^b^ | nd | 0.141±0.008^a^ |
| 13.5 | Undecane | 0.242±0.022 | nd | nd | nd |
| 14.5 | 5,5,6-Trimethylhept-3-en-2-one | 0.185±0.017^b^ | 0.077±0.006^a^ | nd | nd |
| 14.9 | (E)-2-Nonenal | 0.883±0.057^b^ | 0.939±0.056^b^ | 0.873±0.048^b^ | 0.531±0.053^a^ |
| 15.2 | Benzoic acid ethyl ester | 0.925±0.083^b^ | nd | nd | 0.130±0.008^a^ |
| 15.8 | Dodecane | 0.432±0.039^d^ | 0.212±0.017^c^ | 0.157±0.011^b^ | 0.041±0.002^a^ |
| 15.9 | Decanal | 0.138±0.012^b^ | nd | 0.444±0.031^c^ | 0.077±0.005^a^ |
| 16.0 | 2-(ethylthio)propanoic acid ethyl ester | nd | nd | nd | 0.088±0.005 |
| 16.1 | (E,E)-2,4-Nonadienal | 0.872±0.060^d^ | 0.177±0.011^a^ | 0.399±0.021^c^ | 0.227±0.026^b^ |
| 16.2 | Sorbic acid vinyl ester | nd | nd | nd | 0.115±0.007 |
| 16.5 | Benzothiazole | nd | nd | nd | 0.036±0.002 |
| 16.8 | Benzeneacetic acid ethyl ester | nd | nd | 0.145±0.012^b^ | 0.049±0.003^a^ |
| 17.0 | 4a,5-Dimethylhexahydro-4H-1,3-benzodioxin-4-one | nd | nd | 0.240±0.011 | nd |
| 17.2 | Dec-(2E)-enal | 0.330±0.029 | nd | nd | nd |
| 17.6 | 4,6-dimethyldodecane | 0.379±0.034^a^ | 0.485±0.039^b^ | nd | nd |
| 17.8 | 2-Undecanone | nd | nd | 2.30±0.16 | nd |
| 17.9 | Benzo-2,3-pyrrole | nd | nd | nd | 0.094±0.006 |
| 18.0 | Tridecane | 0.304±0.027^c^ | 0.198±0.016^b^ | 0.149±0.011^a^ | nd |
| 18.3 | Deca-(2E,4E)-dien-1-ol | 0.345±0.031 | nd | nd | nd |
| 19.3 | n-Decanoic acid | 0.069±0.006^a^ | 0.581±0.046^c^ | 0.115±0.008^b^ | nd |
| 19.6 | cis-4,5-Epoxy-(E)-2-decenal | nd | nd | 0.111±0.007 | nd |
| 19.7 | trans-4,5-Epoxy-(E)-2-decenal | nd | nd | 0.570±0.031^b^ | 0.081±0.009^a^ |
| 20.0 | Tetradecane | 0.655±0.059^d^ | 0.555±0.044^c^ | 0.468±0.033^b^ | 0.095±0.009^a^ |
| 20.3 | 2,4,7,9-Tetramethyl-5-decyn-4,7-diol | 0.250±0.023^a^ | 0.301±0.024^b^ | 0.275±0.019^a,b^ | nd |
| 21.2 | 2,6,10-trimethyldodecane, | 0.149±0.013^c^ | nd | 0.095±0.007^b^ | 0.031±0.003^a^ |
| 22.2 | 2,4-bis(1,1-dimethylethyl)phenol | nd | 0.150±0.012^b^ | 0.092±0.006^a^ | nd |
| 22.7 | Pentadecane | 0.080±0.007^b^ | 0.161±0.013^d^ | 0.099±0.007^c^ | 0.029±0.003^a^ |
| LUHS29 – treated with *Pediococcus acidilactici* LUHS29; LUHS245 – treated with *Liquorilactobacillus uvarum* LUHS245; LUHS122 – treated with *Lactoplantibacillus plantarum* LUHS122; RT – retention time, in minutes; Mean values (n = 3) ± standard error (SE).  ^a-c^ Means with different letters in column are significantly different (*p* ≤ 0.05). nd – not detected. | | | | | |
